# A systematic review of de-escalation strategies for redeployed staff and repurposed facilities in COVID-19 intensive care units (ICUs) during the pandemic

**DOI:** 10.1016/j.eclinm.2022.101286

**Published:** 2022-02-07

**Authors:** Sigrún Eyrúnardóttir Clark, Georgia Chisnall, Cecilia Vindrola-Padros

**Affiliations:** Rapid Research, Evaluation and Appraisal Lab (RREAL), Department of Targeted Intervention, University College London, London W1W 7TY, UK

**Keywords:** De-escalation, COVID-19, Redeployment, ICUs

## Abstract

**Background:**

Intensive care units (ICUs) experienced a surge in patient cases during the COVID-19 pandemic. Demand was managed by redeploying healthcare workers (HCWs) and restructuring facilities. The rate of ICU admissions has subsided in many regions, with the redeployed workforce and facilities returning to usual functions. Previous literature has focused on the escalation of ICUs, limited research exists on de-escalation. This study aimed to identify the supportive and operational strategies used for the flexible de-escalation of ICUs in the context of COVID-19.

**Methods:**

The systematic review was developed by searching eight databases in April and November 2021. Papers discussing the return of redeployed staff and facilities and the training, wellbeing, and operational strategies were included. Excluded papers were non-English and unrelated to ICU de-escalation. Quality was assessed using the mixed methods appraisal tool (MMAT) and authority, accuracy, coverage, objectivity, date, and significance (AACODS) checklist, findings were developed using narrative synthesis and thematic analysis.

**Findings:**

Fifteen papers were included from six countries covering wellbeing and training themes encompassing; time off, psychological follow-up, gratitude, identification of training needs, missed training catch-up, and continuation of ICU and disaster management training. Operational themes included management of rotas, retainment of staff, division of ICU facilities, leadership changes, traffic light systems, and preparation for re-expansion.

**Interpretation:**

The review provided an overview of the landscape of de-escalation strategies that have taken place in six countries. Limited empirical evidence was available that evaluated the effectiveness of such strategies. Empirical and evaluative research from a larger array of countries is needed to be able to make global recommendations on ICU de-escalation practices.

**Research in context**

**Evidence before this study**

Previous research has explored the expansion of intensive care units (ICUs) during the pandemic, to address the increase in patient demand, and the strategies for the redeployment of staff, including the integration of training and wellbeing support. This review searched eight databases of peer-reviewed and grey literature in April and November 2021, with no limitations in relation to geographic location, but limited to articles published in English. Detailed search criteria were developed around the phrases ‘COVID-19′, ‘de-escalation’, ‘ICU response’, ‘redeployment’ and ‘healthcare workforce’, and the quality of included articles was assessed using the mixed methods appraisal tool (MMAT) and authority, accuracy, coverage, objectivity, date, and significance (AACODS) checklist.

**Added value of this study**

To our knowledge, this is the first systematic review of the evidence on the de-escalation of ICUs in the context of the COVID-19 pandemic. The key operational approach in relation to maintaining flexibility for future surges was to use a traffic light or phased return system for both the workforce and the facilities, as it would allow for a quick return to redeployment, if needed. The key supportive strategies have focused on the wellbeing and the training needs of the returning redeployed workforce, which included ensuring that staff received time off to rest and recuperate. These strategies also entailed monitoring and supporting the long-term mental health of staff ensuring staff received recognition and gratitude for their service; identifying training needs in the trainee healthcare workforce and catching up on any missed training. The most relevant training strategy in relation to preparation for future surges of COVID-19 was to continue with ICU and disaster preparedness training and practices.

**Implications of all the available evidence**

This review has served as a first step to map the available evidence on the strategies that are currently being used for the de-escalation of ICUs in healthcare settings from six countries. To enhance the field of planning for de-escalation, further evidence should be collected from a wider range of healthcare settings globally. This would enable the further sharing of experiences, allowing healthcare leaders and policymakers to identify strategies that could be adapted to their local setting.

Alt-text: Unlabelled box

## Introduction

Just over 200 million cases and 4.4 million deaths had been reported worldwide due to severe acute respiratory syndrome coronavirus 2 (SARS-CoV-2) by August 2021.^1^ COVID-19 has placed an unprecedented demand on intensive care units (ICUs) around the world. As a result, health systems have faced challenges in sourcing the care providers and resources needed to provide intensive care.^2^ Building new ICU facilities within hospitals has been an unpopular option as the excess ICU space would become inefficient following the pandemic.^3^ Likewise, the limited pool of ICU qualified healthcare workers (HCWs) made it difficult to source new staff to fulfil the intensive care responsibilities.^4^ Most healthcare settings have, therefore, had to make do with the limited resources available in the time-sensitive conditions to restructure existing facilities and redeploy the existing healthcare workforce to the ICUs. Many settings expanded ICU facilities by developing temporary ICUs in operating theatres that were not in use as elective procedures had been cancelled, by converting paediatric ICUs (PICU) into adult ICUs as the infection had not affected children as severely as adults, or by outsourcing patients to private healthcare providers.^5^, ^6^, ^7^, ^8^ To fulfil the workforce requirements, medical professionals were redeployed from the areas of care that were not in high demand such as surgical departments and PICUs. Additionally former HCWs and trainee HCWs were redeployed.^5^^,^^6^^,^^8^, ^9^, ^10^, ^11^ Elective surgery and other speciality training opportunities were cancelled to allow the redeployment of healthcare professionals, especially trainee doctors.^11^, ^12^, ^13^, ^14^

The first peak of COVID-19 cases and ICU admissions has come and gone for numerous countries. By August 2020 many countries had got through the first peak of ICU admissions whilst suffering considerably, and faced the prospect of future surges.^15^ With the decline in ICU patients, many of the redeployed workforce and temporary ICUs could return to usual functions, and numerous publications have documented how these processes have occurred in different hospital settings.^8^^,^^16^, ^17^, ^18^, ^19^, ^20^, ^21^, ^22^, ^23^, ^24^, ^25^, ^26^, ^27^, ^28^, ^29^ Since the initial decline, many countries have faced recuring waves of COVID-19 infections and ICU admissions.^15^ This repetitive surge in need for ICU capacity followed by de-escalation is likely to continue for some time whilst variants of SARS-CoV-2 continue to circulate throughout the globe.^30^ Although mass vaccination efforts are preventing a large proportion of COVID-19 related hospital admissions, they are not 100% effective.^31^ Additionally, there are still large populations of the world that remain unvaccinated such as children, individuals unable to receive the vaccination due to health reasons, vaccine hesitant individuals, and populations from Low- and Middle-Income countries (LMICs) that have yet to receive the vaccination doses.^32^, ^33^, ^34^, ^35^, ^36^

The purpose of this systematic review was to systematically review the strategies that have been used to de-escalate the ICU response and return redeployed staff and facilities to their usual role. The review focuses on the strategies to provide wellbeing and training support, and the operational strategies to manage the return of the workforce and facilities in contexts that are shaped with the potential need to escalate services due to future surges. Identifying these strategies is an important starting point to promote research into the evaluation of reported strategies globally. Once evaluation research is available it would then be possible to make de-escalation recommendations for recurrent surges of COVID-19.

The review was guided by the following research questions: What are the mechanisms developed to support the wellbeing of the healthcare workforce following redeployment periods? What are the training strategies recommended to support healthcare staff as they return from redeployment? What operational strategies have been suggested to manage the return of the healthcare workforce from redeployed areas? What operational strategies have been established to manage the de-escalation of intensive care facilities?

## Methods

The review was designed as a systematic review capable of capturing the emerging evidence on de-escalation strategies of ICUs following COVID-19 surges. The review was developed following the Preferred Reporting Items for Systematic Reviews and Meta-Analyses (PRISMA) 2020 statement and following the systematic review guide shared by Tricco et al.^37^^,^^38^ A protocol was developed prior to conducting the research and was accepted by PROSPERO in March 2021 and has since been updated to reflect the change in focus from general epidemics to the COVID-19 pandemic specifically (registration number - CRD42021244900).^39^

### Search strategy

Following the creation of the research questions, an initial list of search items was developed using the PICO framework.^40^^,^^41^ Key terms were selected with librarian input and by reviewing prior reviews on COVID-19.^42^ The search terms and Boolean operators used for each database can be found in Appendix 1. Databases containing peer-reviewed and grey literature were then searched using the relevant search criteria between 13 and 16 April 2021 and a second wave of searches on 18 November 2021. The databases containing peer reviewed literature included: MEDLINE/Ovid, CINAHL Plus/EBSCOhost, PsycINFO/Ovid, and Web of Science. The grey literature databases included: Health Management Information Consortium, TRIP, NICE Evidence Search, and medRxiv.

### Eligibility criteria

The eligibility criteria can be found in Table 1 below (for additional information, see Appendix 1).

**Table1:** Inclusion and exclusion criteria.

Inclusion	Exclusion
Publications up to November 2021	Non-English language
Redeployed HCWs of all specialisms returning from ICUs	HCWs redeployed to other areas outside of ICUs
Wellbeing support for the staff following redeployment	Escalation of ICU only, with no mention of de-escalation
Training strategies for redeployed HCWs returning from ICUs	Broad de-escalation of the whole hospital rather than the ICU specifically
Operational strategies to manage the de-escalation of the workforce	
Operational strategies to manage the de-escalation of ICU facilities	
ICUs globally that have been re-structured during COVID-19	

### Selection process

The search results were imported to Rayyan.^43^ Following the initial screening by title and abstract, a second researcher cross checked 10% of exclusions against the eligibility criteria. The interrater reliability was then tested using a percentage of agreement rate where a percentage above 80% was deemed acceptable.^44^ The agreement rate was 98% for the first wave of screening and 100% for the second wave of screening. Any disagreements in the choice of exclusions between the two researchers were discussed until resolved as advised by the Cochrane handbook.^45^

Following the title and abstract screening, the remaining publications that met the inclusion criteria were imported into an Excel document and the full text was screened for eligibility. The publications identified during the review were also added to the full text screening process. The second reviewer cross-checked 10% of exclusions, using the same percentage agreement rate threshold (agreement rate 89% for first wave and 100% for the second wave of screening), and any discrepancies were discussed until resolved. The references of the final list of included publications were also screened to identify any additional relevant articles.

### Data extraction

The data from the included publications were extracted by using a data extraction form developed in Excel using a pre-defined list based on themes identified in the screening process (see Appendix 2). The extracted study details included information on study design, study population, study setting, and study methodology. The main experiences collected from the publications included wellbeing support initiatives, training strategies, and operational strategies for the workforce and the ICU facilities. Additional data on limitations were also collected.

### Quality assessment

The mixed methods appraisal tool (MMAT) was used for qualitative, quantitative, and mixed methods studies to assess their quality, reliability, and relevance.^46^ See Appendix 3 for the MMAT.^47^, ^48^, ^49^ To assess the authority, accuracy, coverage, objectivity, date, and significance (AACODS) of the grey literature, the AACODS checklist was used.^50^^,^^51^ See Appendix 4 for the AACODS checklist.^52^^,^^53^ A second reviewer then cross checked 10% of the critical appraisal scores and justifications of scores. Any discrepancies between the MMAT and AACODS scores were discussed until resolved.

### Data synthesis

Narrative synthesis was conducted based on the methodology used by Popay et al.^54^ to summarise the key characteristics of each study and to highlight the differences and similarities between the studies. Thematic synthesis was then conducted based on the methodology advised by Braun and Clarke.^55^ To conduct the synthesis, meaningful qualitative data from the articles were highlighted in a deductive manner, so only text relevant to the four sub-research questions were included. The categories were then grouped into the four core themes.

## Results

### Study selection

**Figure. 1:**
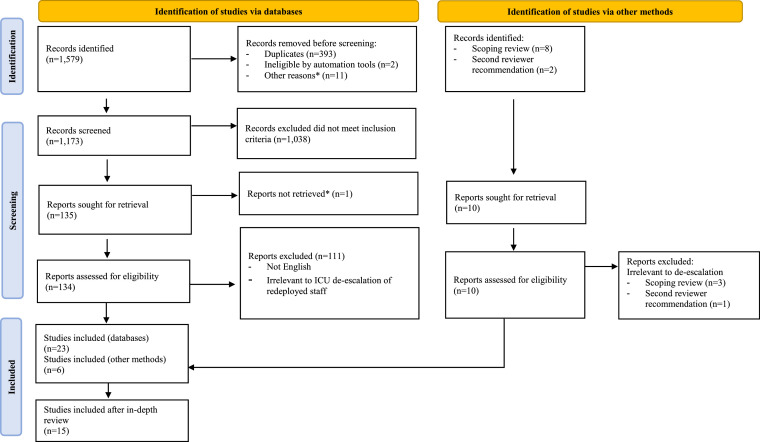
PRISMA 2020 flow diagram of systematic review results.^36^ * Unable to access publications due to restricted access to journals or limited citation information.

The screening and selection process can be seen in Figure. 1.^37^ The search of eight databases returned 1579 records, the removal of inaccessible or duplicated publications resulted in 1173 records. Screening of publications based on title and abstract resulted in the exclusion of 1038 records. Screening of the remaining papers based on full text resulted in the exclusion of 111 records. The reasons for exclusion were due to the content being irrelevant to ICU de-escalation and staff redeployment or were not in English.

During the data extraction process, an in-depth review of the 29 papers resulted in 14 papers being excluded as the content was related to broad hospital responses of de-escalation rather than ICU specific responses or was related to existing staff in ICUs rather than redeployed staff. The screening of references of the final 15 publications did not result in additional publications for inclusion.

### Study characteristics

A summary of the key characteristics of the included papers can be found in Table 2. Of the 15 papers included, two were based on empirical data, ten were based on data from case studies, commentary articles or letters to the editor,^16^, ^17^, ^18^, ^19^, ^20^, ^21^, ^22^^,^^24^^,^^29^ and three were guidelines on how to manage the de-escalation of ICUs.^25^^,^^26^^,^^28^ The included papers were from the UK (*n* = 6), the USA (*n* = 4), Singapore (*n* = 2), China (*n* = 1), Iran (*n* = 1), and Australia (*n* = 1).

**Table 2:** Summary of study characteristics and quality scores.

Author	Publication	Location	Profession	Themes	Quality
Faculty of Intensive Care Medicine^25^	Guidelines	NHS, UK	Surgical staff	Operational strategies for workforce and facilities	AACODS: 3/6
Leng et al.^27^	Case study of actions derived from a mixed methods study	Unnamed hospital Wuhan, China The Affiliated Hospital of Qingdao University, Qingdao, China	90 nurses with COVID-19 and ICU experience	Wellbeing and training strategies	AACODS:6/6
McCabe et al.^8^	Empirical study based on secondary data analysis	NHS data, UK	Former, private and trainee medical staff	Operational strategies for workforce and facilities	AACODS: 5.5/6
Schneider et al.^16^	Case study	New York-Presbyterian/Weill Cornell Medical center, New York, USA	Neuroscience ICU and non-ICU nurses	Training strategies	AACODS: 4.5/6
Whitby et al.^22^	Commentary article	Alder Hey Children's Hospital, Liverpool, UK	Trainees	Operational strategies for workforce	AACODS: 6/6
Caroselli^24^	Case study	Veterans Affairs New York harbour Healthcare System, New York, USA	ICU and non-ICU nurses	Wellbeing and training strategies	AACODS: 4.5/6
Yau et al.^29^	Letter to the Editor	Tan Tock Seng Hospital and National Centre of Infectious Diseases, Singapore	N/A	Operational strategies for facilities	AACODS: 4/6
Lord et al.^17^	Case study	NYU Langone-Brooklyn Hospital, New York, USA	Neurology staff	Operational strategies for workforce and facilities	AACODS: 5.5/6
Panayiotou et al.^18^	Case study	Kings College Hospital, London, UK	Radiologist trainees	Training, wellbeing, and operational strategies for workforce	AACODS: 5.5/6
Doyle et al.^20^	Case study	John Radcliffe Hospital, Oxford, UK	Medical staff	Operational strategies for workforce	AACODS: 5.5/6
Lum et al.^19^	Commentary article	National University Hospital, Singapore	Medical staff	Training strategies and operational strategies for workforce and facilities	AACODS: 5.5/6
Poortaghi et al.^23^	Qualitative study	Various hospitals, Iran	Nurses based on interviews from 15 nurse managers	Wellbeing strategies	MMAT: 4/5
Price et al.^26^	Editorial based on guidelines	NHS, UK	Surgical staff	Training, wellbeing, and operational strategies for workforce	AACODS: 5.5/6
Marshall et al.^28^	Guidelines	Australia	Medical staff	Wellbeing and operational strategies for workforce	AACODS: 5/6
Shaparin et al.^21^	Case study	Montefiore Medical Centre, New York, USA	Anaesthesiology staff	Operational strategies for facilities	AACODS: 4.5/6

### Quality assessment

An overview of the quality assessment scores can also be found in Table 2, with the detailed results in Appendices 3 and 4. There were 13 publications that met 4.5 or more of the AACODS criteria or 4 or more of the MMAT criteria, which categorised them as high quality. A second reviewer reviewed 10% of the quality assessment scores and did not have any disagreements.

### Thematic synthesis

The key findings from the articles included in the review could be organised across the four categories presented in Table 3.

**Table 3:** Summary of findings.

Themes	Categories
Wellbeing strategies	Provide time off to returning redeployed staff Monitor the mental health of the returning staff over the long-term Administer post-redeployment interviews to returning staff Show returning staff respect and gratitude
Training strategies	Ensure returning trainees catch up on missed training Supervise returning trainees to identify training needs Continue to train redeployed staff from non-ICU specialities in ICU practices Continue training in disaster management for staff that may face future redeployment
Operational strategies for healthcare workforce	Change the staffing rotas to enable the return to usual roles or to account for time off Retain staff to allow for time off or for backlog of care needs, or for future surges Continue outsourcing to private providers to keep staff volumes high Administer a traffic light system or stepwise approach to returning staff to usual roles
Operational strategies for facilities	Division of ICU facilities into COVID-19-positive and -negative wards Changes in leadership of teams that managed the COVID-19 ICUs Traffic light system or stepwise approach to return facilities back to usual functions Ensure facilities are on standby for re-expansion of ICUs

#### Wellbeing strategies

A common strategy implemented to improve the wellbeing of the returning redeployed staff was to provide time off to rest and recuperate.^18^^,^^23^^,^^26^, ^27^, ^28^ Leng et al.^27^ shared that following the redeployment period, the nurses received a three week holiday and Panayiotou et al.^18^ shared a similar experience that returning trainees were encouraged to take vacation leave. Poortaghi et al.^23^ shared that nurse managers in various hospitals in Iran had promised the redeployed nurses’ access to annual leave once the surge of patients had minimised. Price et al.^26^ and Marshall et al.^28^ made recommendations for the UK and Australia, respectively, that ahead of resuming normal activities, redeployed staff should get access to leave or reduced hours.

A concern among hospitals were the long-term mental health effects of working in ICUs during the pandemic. Panayiotou et al.^18^ shared that post-redeployment interviews were conducted to identify any needs for mental health support, and likewise Leng et al.^27^ shared that a psychological taskforce followed up with the nurses post-deployment to determine if they required any support or if they had signs of post-traumatic stress disorder (PTSD). There was an overall feeling that HCWs deserved a great deal of respect. Caroselli^24^ shared that gratitude was expressed to the redeployed staff throughout and after the surge through means of personal communication and presentations.

#### Training strategies

A key experience outlined in three of the papers was that redeployed trainees should have their missed training resumed once returned to usual roles.^18^^,^^19^^,^^26^ Price et al.^26^ suggested as surgical activity resumes following de-escalation of ICUs, training needs must be accommodated within all NHS surgical departments. Lum et al.^19^ echoed this and recommended that following the surge, restrictions must be addressed to allow the completion of training for trainees, many of whom were redeployed. Panayiotou et al.^18^ summarised the actions taken at Kings College Hospital whereby the radiology trainees were re-introduced to training and were supervised to identify any educational needs following the redeployment.

HCWs not only required further training in their usual fields, but some requested further training in the ICU field. Schneiderl.^16^ highlighted how non-ICU nurses had vocalised their desire to continue learning about the higher-level skills from their ICU redeployment period. Additional educational activities that took place during the redeployment have also continued in some settings, as shared by Panayiotou et al.,^18^ the multidisciplinary educational meetings between surgical trainees and the ICU team have continued to allow important findings to be discussed.

Many healthcare settings acknowledged that future surges of COVID-19 and other epidemics are expected, so recommended the training of staff in disaster management should be continued following redeployment to allow for flexibility for future threats. This was recommended by Leng et al.,^27^ Caroselli^24^ made a similar recommendation, suggesting that staff should continue regular redeployment rotations to maintain the skills that are needed in response to an emergency.

#### Operational strategies for returning the HCWs

Many of the hospitals facilitated the return of the redeployed staff to their usual departments through updates to rotas.^17^^,^^18^^,^^22^^,^^25^ Whitby et al.^22^ shared how a process had been prepared to de-escalate the COVID-19 rota to enable the return of redeployed trainees. Lord et al.^17^ described that once the number of COVID-19 patients had subsided, the staff returned to their usual roles. Panayiotou et al.^18^ shared the same experience of trainees returning to their usual department and that the rota coverage had been reduced during de-escalation. The Faculty of Intensive Care Medicine et al.^25^ recommended that, once surgical departments were ready to return to usual function, the rotas and intensive care staffing ratios should be restored to normal staffing levels.

In addition to adapting rotas to allow the return to usual roles, both Price et al.^26^ and Marshall et al.^28^ recommended that rotas should factor in time off for the returning staff. In doing so, the institutions must recognise that there will be a reduction in staff availability^26^ so a portion of the redeployed workforce in ICUs should be reserved to allow for this.^28^ Similarly, McCabe et al.^8^ recommended that redeployed former and private HCWs should be retained in the ICUs following the surge to support with caring for the backlog of intensive care need. Lum et al.^19^ shared the experience that despite the surge of patients abating, the redeployed staff remained on standby for future surges.

Some of the papers illustrated a step-down or traffic light approach to the de-escalation of ICUs.^20^^,^^25^ The Faculty of Intensive Care Medicine.^25^ recommended using a traffic light system in which checkpoint assessments were used to determine whether individual NHS hospitals were ready to return redeployed staff to usual roles in surgery departments. Doyle et al.^20^ shared the experience of returning redeployed staff to usual roles gradually in a step-down manner which gave them the flexibility to step-up redeployment should the ICUs surge again.

#### Operational strategies for de-escalating ICU facilities

Some of the papers documented similar approaches to the traffic light system to manage the return of redeployed staff, for de-escalating the ICU facilities.^25^^,^^29^ Yau et al.^29^ discussed, how the ICU facilities were de-escalated in 3 phases. The first two phases included separating the ICUs between two healthcare settings, so only one remained open for COVID-19 admissions. The final phase included passing all triaging responsibility back to the centre still taking COVID-19 cases which was previously in the hands of the outbreak ICU headquarters setup during the surge. The Faculty of Intensive Care Medicine^25^ also shared recommendations on how to gradually return ICU facilities based on the three broad traffic light checkpoints, with the final checkpoint recommending the division of COVID-19-positive and -negative ICU beds. This approach for separating the ICU was also experienced in the setting described by Lord et al.^17^

A common theme in the management of ICU facilities is that some of the healthcare settings were wary of future COVID-19 surges. Shaparin et al.^21^ discussed how the anaesthesiology team were planning to prepare for the future closure of Ambulatory Surgery Centre to enable its transformation into ICUs should a resurgence of COVID-19 occur. Yau et al.^29^ and Lum et al.^19^ both shared that the teams kept oversight of ICU supplies and equipment, and the team in the Lum et al. paper ring-fenced 10% of the remaining ICU beds. All these approaches were implemented to ramp up ICU capacity if needed in response to future COVID-19 surges.

## Discussion

This review has identified the supportive and operational strategies that have been implemented and documented to date to manage the de-escalation of ICUs in certain countries where COVID-19 admissions have experienced a decline.

The key supportive strategies focussed on the wellbeing and the training needs of the returning redeployed workforce. Wellbeing mechanisms concentrated on ensuring that the staff received time off to rest and recuperate; they also entailed monitoring and supporting the long-term mental health of staff; and ensuring the staff received recognition and gratitude for their service. The most obvious training strategies were to identify training needs of the trainee HCWs and to catch up on any missed training. The most relevant training strategy in relation to preparation for future surges of COVID-19 was to continue with ICU and disaster preparedness training and practices.

De-escalation strategies also included operational approaches to manage the return of the workforce and facilities. The key operational approach in relation to maintaining flexibility for future surges was the plan to use a traffic light or phased return system for both the workforce and the facilities, as it would allow for a quick return to redeployment if needed. Maintaining oversight of ICU supplies, ringfencing ICU beds, and preparing non-COVID-19 wards for closure were other plans to ramp up ICU capacity, if needed. Other operational strategies included updating staff rotas to return staff to usual roles or to maintain a portion of the workforce to facilitate vacations and to deal with the backlog of critical care needs. It was also documented that ICUs were separated into COVID-19-positive and -negative beds to focus on care needs outside of COVID-19. In terms of management, it was suggested that control should be returned from a central overarching body to the specific centre managing the COVID-19 cases.

The research included in this review focused mainly on the de-escalation of redeployed HCWs in comparison to the de-escalation of facilities. This contrasts the heavy reporting of strategies used to escalate facilities during the COVID-19 surge.^5^, ^6^, ^7^, ^8^

Similarities exist between the wellbeing strategies that have been described in this review and strategies that have been implemented following other emergencies. In the aftermath of Hurricane Katrina, plans were made to ensure mental health services were available to nurses, due to the adverse emotional outcomes they had reported.^56^^,^^57^ It was also recommended to show recognition and gratitude to nurses in Taiwan following the 2003 SARS epidemic.^58^ Jones et al.^59^ shared research on the post-deployment method within the armed forces, which allowed staff to unwind and relax after their deployment period. This approach was associated with a decrease in the incidence of PTSD.

Research by Chang et al. and Shiao et al. found an increase in nurse resignations and staff turnover following the SARS outbreak due ongoing feelings of stress.^60^^,^^61^ Similar findings were found by the Institute for Public Policy Research think tank in relation to COVID-19, where survey results found that 1 in 4 NHS staff in the UK were more likely to resign from their positions.^62^^,^^63^ These findings strengthen the purpose of the wellbeing strategies discussed in this review, to ensure the healthcare workforce are present and mentally supported to deal with future surges of COVID-19 and to deal with the backlog of care needs.

The plans to ensure that ICU and disaster preparedness training and practices continue is not a novel idea. Following the SARS epidemic, research by Thomas et al. and Lam et al. concluded that training in disaster management and emergency preparedness should continue to prepare HCWs for future epidemics.^64^^,^^65^ However, more recent research has found that, prior to the COVID-19 pandemic, the level of disaster preparedness and ICU training in healthcare settings was relatively poor.^56^^,^^66^, ^67^, ^68^, ^69^, ^70^ Research by Liu et al. highlighted that nurses who were working during the SARS epidemic and again during the COVID-19 pandemic required further disaster management training to cope with the COVID-19 pandemic despite the SARS experience.^71^ This emphasises the importance of ensuring training in disaster management that follows the de-escalation of ICUs, as it appears not to have been maintained following previous epidemics.

Maintaining flexibility in the operational management of staff and facilities to adapt to future ICU demands has been recommended previously following the SARS outbreak and numerous influenza outbreaks.^72^^,^^73^ During non-pandemic times, maintaining all the excess ICU staff and facilities would be a waste of resources.^3^^,^^74^ So, gradual approaches that enable return to usual functions whilst maintaining flexibility to expand ICU capacity in times of need would be extremely useful.

There is a difference between the previous contexts described above and the context of the COVID-19 pandemic. This pandemic is unique in the ongoing emergence of new waves of infection across the world. The new waves of infections have meant that the healthcare workforce have had to be agile with de-escalating and re-escalating the ICU response in terms of staffing, restructuring facilities, and ensuring staff receive the necessary training and wellbeing support to cope with the recurrent demands on ICU facilities. This highlights the pressing need to develop strategies of de-escalation that are flexible for re-escalation, which this review has attempted to identify.

The primary limitation of the review is the lack of representativeness as most of the included articles were from the UK and the US. There were no reports from Africa, Latin America, Europe, and most of Asia. The reason for the limited geographical scope could be due to limiting articles to those published in English. Other explanations for the limited de-escalation literature from LMICs could be because healthcare settings in these regions are still dealing with high numbers of hospitalisations (and are focussed on the escalation, rather than the de-escalation of ICUs) or have not had time and resources to develop reports and peer-reviewed articles on de-escalation. The strategies discussed in the review are, therefore, not representative of all healthcare settings globally and may not be feasible in different regions of the globe.

This review set out to identify ICU de-escalation strategies from empirical data and from grey literature. However, limited empirical data were identified and the majority of data were based on recounts from hospital departments on the de-escalation strategies implemented locally.

This review has served as a first step to map the available evidence on the strategies that are currently being used for the de-escalation of ICUs. To further enhance the field of planning for de-escalation, strategies in the form of guidelines, case studies and plans that have been implemented, should be collected from a wider range of settings. This would enable the sharing of experiences across the globe and the identification of what works, allowing healthcare leaders and policymakers to identify strategies that could be adapted to their settings.

## Contributors

SEC led the study design, data collection, data analysis, and data interpretation, and contributed to the drafting and revision of the manuscript. CV-P led the study conception, drafting and revision of the manuscript, and contributed to the study design, data analysis, and data interpretation. GC contributed to the study conception and data analysis. All authors were responsible for the raw data associated with the study. CVP and SC made the decision to submit the manuscript for publication.

### Data sharing statement

Datasets utilized in this study can be accessed upon reasonable request. All datasets within the study are available within the article or supplementary material.

## Declaration of interests

None declared.

## Appendix Supplementary materials

Image, application 1
