# Supplementary material for: A systematic review of de-escalation strategies for redeployed staff and repurposed facilities in COVID-19 intensive care units (ICUs) during the pandemic
Source: eClinicalMedicine. 2022 Feb 7;44:101286. doi: 10.1016/j.eclinm.2022.101286 (PMC8820730; doi:10.1016/j.eclinm.2022.101286)
Supplement: Supplementary file 1 [file mmc1.docx]

**Supplementary material**

**Appendix 1 – Search criteria** *(searched 13 April and 18 November 2021)*

*Appendix 1.1 – Search criteria: MEDLINE/OVID*

| **Search number** | **Search terms** |
| --- | --- |
| S1 | exp Coronavirus/ |
| S2 | exp Coronavirus Infections/ |
| S3 | exp Pneumonia, Viral/ |
| S4 | epidemics/ or pandemics/ |
| S5 | ((corona* or corono*) adj1 (virus* or viral* or virinae*)).ti,ab. |
| S6 | (coronavirus* or coronovirus* or coronavirinae* or CoV).ti,ab. |
| S7 | ("2019-nCoV" or "2019nCoV" or "nCoV2019" or "nCoV-2019" or "COVID-19" or "COVID19" or "CORVID-19" or "CORVID19" or "WN-CoV" or "WNCoV" or "HCoV-19" or "HCoV19" or "2019 novel*" or "Ncov" or "n-cov" or "SARS-CoV-2" or "SARSCoV-2" or "SARSCoV2" or "SARS-CoV2" or "SARSCov19" or "SARS-Cov19" or "SARSCov-19" or "SARS-Cov-19" or "Ncovor" or "Ncorona*" or "Ncorono*" or "NcovWuhan*" or "NcovHubei*" or "NcovChina*" or "NcovChinese*" or "SARS2" or "SARS-2" or "SARScoronavirus2" or "SARS-coronavirus-2" or "SARScoronavirus 2" or "SARS coronavirus2" or "SARScoronovirus2" or "SARS-coronovirus-2" or "SARScoronovirus 2" or "SARS coronovirus2").ti,ab. |
| S8 | (respiratory* adj2 (symptom* or disease* or illness* or condition*) adj5 (Wuhan* or Hubei* or China* or Chinese* or Huanan*)).ti,ab. |
| S9 | (("seafood market*" or "food market*" or "pneumonia*") adj5 (Wuhan* or Hubei* or China* or Chinese* or Huanan*)).ti,ab. |
| S10 | ((outbreak* or wildlife* or pandemic* or epidemic*) adj5 (Wuhan* or Hubei* or China* or Chinese* or Huanan*)).ti,ab. |
| S11 | "severe acute respiratory syndrome*".ti,ab. |
| S12 | or/1-11 |
| S13 | exp Critical Care/ |
| S14 | intensive care units/ or coronary care units/ or recovery room/ or respiratory care units/ |
| S15 | "ICU".ti,ab. |
| S16 | "ITU".ti,ab. |
| S17 | "intensive care".ti,ab. |
| S18 | "critical care".ti,ab. |
| S19 | "respiratory care unit*".ti,ab. |
| S20 | Tertiary Healthcare/ |
| S21 | hospitals/ or hospitals, general/ or hospitals, high-volume/ or hospitals, low-volume/ or hospitals, public/ or hospitals, teaching/ or hospitals, urban/ or secondary care centers/ or tertiary care centers/ |
| S22 | hospital*.ti,ab. |
| S23 | "tertiary care".ti,ab. |
| S24 | "secondary care".ti,ab. |
| S25 | or/13-24 |
| S26 | exp "personnel staffing and scheduling"/ or return to work/ |
| S27 | Surge Capacity/ |
| S28 | exp Disaster Planning/ |
| S29 | "de-escalat*".ti,ab. |
| S30 | "relaxation*".ti,ab. |
| S31 | "return* to work".ti,ab. |
| S32 | "reset".ti,ab. |
| S33 | "new normal".ti,ab. |
| S34 | "post pandemic".ti,ab. |
| S35 | "pre pandemic".ti,ab. |
| S36 | "mitigat*".ti,ab. |
| S37 | "curtail*".ti,ab. |
| S38 | or/26-37 |
| S39 | exp Health Personnel/ |
| S40 | Health Workforce/ |
| S41 | "health* personnel".ti,ab. |
| S42 | "health* staff".ti,ab. |
| S43 | "health* workforce".ti,ab. |
| S44 | "health* worker*".ti,ab. |
| S45 | "medical staff".ti,ab. |
| S46 | "health* professional*".ti,ab. |
| S47 | "redeploy*".ti,ab. |
| S48 | or/39-47 |
| S49 | 12 and 25 and 38 and 48 |

*Appendix 1.2 – Search criteria: CINAHL Plus/EBSCOhost*

| **Search number** | **Search terms** |
| --- | --- |
| S1 | (MH “Coronavirus+”) |
| S2 | (MH "Coronavirus Infections+") |
| S3 | (MH "Pneumonia, Viral") |
| S4 | (epidemic* or pandemic*or outbreak*) |
| S5 | (coronavirus* or coronovirus* or coronaviral* or coronoviral* or coronavirinae* or coronovirinae*) |
| S6 | ("2019-nCoV" or "2019nCoV" or "nCoV2019" or "nCoV-2019" or "COVID-19" or "COVID19" or "CORVID-19" or "CORVID19" or "WN-CoV" or "WNCoV" or "HCoV-19" or "HCoV19" or "2019 novel*" or "Ncov" or "n-cov" or "SARS-CoV-2" or "SARSCoV-2" or "SARSCoV2" or "SARS-CoV2" or "SARSCov19" or "SARS-Cov19" or "SARSCov-19" or "SARS-Cov-19" or "Ncovor" or "Ncorona*" or "Ncorono*" or "NcovWuhan*" or "NcovHubei*" or"NcovChina*" or "NcovChinese*" or "SARS2" or "SARS-2" or "SARScoronavirus2" or "SARS-coronavirus-2" or "SARScoronavirus 2" or "SARS coronavirus2" or "SARScoronovirus2" or "SARS-coronovirus-2" or "SARScoronovirus 2" or "SARS coronovirus2") |
| S7 | CoV |
| S8 | ("respiratory* symptom*"or "respiratory* disease*"or "respiratory* illness*" or "respiratory condition*") AND (Wuhan* or Hubei* or China* or Chinese* or Huanan*) |
| S9 | (("seafood market*" or "food market*" or "pneumonia*") AND (Wuhan* or Hubei* or China* or Chinese* or Huanan*)) |
| S10 | ((outbreak* or wildlife* or pandemic* or epidemic*) AND (Wuhan* or Hubei* or China* or Chinese* or Huanan*) |
| S11 | "severe acute respiratory syndrome*" |
| S12 | S1 OR S2 OR S3 OR S4OR S5 OR S6 OR S7 ORS8 OR S9 OR S10 OR S11 |
| S13 | (MH "Critical Care+") |
| S14 | (MH "Coronary Care Units") OR (MH "Intensive Care Units") OR (MH "Respiratory Care Units") |
| S15 | ICU |
| S16 | ITU |
| S17 | “intensive care” |
| S18 | “critical care” |
| S19 | “respiratory care unit” |
| S20 | (MM "Tertiary HealthCare") |
| S21 | (MH "Hospitals") OR (MH "Hospitals, Public") OR (MH "Hospitals, Special")OR (MH "Hospitals, Urban") |
| S22 | hospital* |
| S23 | “tertiary care” |
| S24 | “secondary care” |
| S25 | S13 OR S14 OR S15 OR S16 OR S17 OR S18 OR S19 OR S20 OR S21 OR S22 OR S23 OR S24 |
| S26 | (MH "Personnel Staffing and Scheduling+") |
| S27 | (MH "Disaster Planning+") |
| S28 | (MH "Health Facility Planning+") |
| S29 | de-escalat* |
| S30 | relaxation* |
| S31 | “return* to work” |
| S32 | reset |
| S33 | “new normal” |
| S34 | “post pandemic” |
| S35 | “pre pandemic” |
| S36 | mitigat* |
| S37 | curtail* |
| S38 | S26 OR S27 OR S28 OR S29 OR S30 OR S31 OR S32 OR S33 OR S34 OR S35 OR S36 OR S37 |
| S39 | (MH "Health Personnel+") |
| S40 | "health* personnel” |
| S41 | “health* staff" |
| S42 | “health* workforce" |
| S43 | “health* worker*" |
| S44 | “medical staff" |
| S45 | “health* professional*" |
| S46 | “redeploy*" |
| S47 | S39 OR S40 OR S41 OR S42 OR S43 OR S44 OR S45 OR S46 |
| S48 | S12 AND S25 AND S38 AND S47 |

*Appendix 1.3 – Search criteria: APA PsychINFO/Ovid*

| **Search number** | **Search terms** |
| --- | --- |
| S1 | exp Coronavirus/ |
| S2 | pneumonia/ or exp middle east respiratory syndrome/ |
| S3 | epidemics/ or pandemics/ |
| S4 | ((corona* or corono*) adj1 (virus* or viral* or virinae*)).ti,ab. |
| S5 | (coronavirus* or coronovirus* or coronavirinae* or CoV).ti,ab. |
| S6 | ("2019-nCoV" or "2019nCoV" or "nCoV2019" or "nCoV-2019" or "COVID-19" or "COVID19" or "CORVID-19" or "CORVID19" or "WN-CoV" or "WNCoV" or "HCoV-19" or "HCoV19" or "2019 novel*" or "Ncov" or "n-cov" or "SARS-CoV-2" or "SARSCoV-2" or "SARSCoV2" or "SARS-CoV2" or "SARSCov19" or "SARS-Cov19" or "SARSCov-19" or "SARS-Cov-19" or "Ncovor" or "Ncorona*" or "Ncorono*" or "NcovWuhan*" or "NcovHubei*" or "NcovChina*" or "NcovChinese*" or "SARS2" or "SARS-2" or "SARScoronavirus2" or "SARS-coronavirus-2" or "SARScoronavirus 2" or "SARS coronavirus2" or "SARScoronovirus2" or "SARS-coronovirus-2" or "SARScoronovirus 2" or "SARS coronovirus2").ti,ab. |
| S7 | (respiratory* adj2 (symptom* or disease* or illness* or condition*) adj5 (Wuhan* or Hubei* or China* or Chinese* or Huanan*)).ti,ab. |
| S8 | (("seafood market*" or "food market*" or "pneumonia*") adj5 (Wuhan* or Hubei* or China* or Chinese* or Huanan*)).ti,ab. |
| S9 | ((outbreak* or wildlife* or pandemic* or epidemic*) adj5 (Wuhan* or Hubei* or China* or Chinese* or Huanan*)).ti,ab. |
| S10 | "severe acute respiratory syndrome*".ti,ab. |
| S11 | or/1-10 |
| S12 | exp intensive care/ |
| S13 | ("intensive care unit" or "coronary care unit" or "recovery room" or "respiratory care unit").ti,ab. |
| S14 | "ICU".ti,ab. |
| S15 | "ITU".ti,ab. |
| S16 | "critical care".ti,ab. |
| S17 | "intensive care".ti,ab. |
| S18 | "respiratory care unit*".ti,ab. |
| S19 | "tertiary healthcare".ti,ab. |
| S20 | hospitals/ or hospitals, general/ or hospitals, high-volume/ or hospitals, low-volume/ or hospitals, public/ or hospitals, teaching/ or hospitals, urban/ or secondary care centers/ or tertiary care centers/ |
| S21 | hospital*.ti,ab. |
| S22 | "tertiary care".ti,ab. |
| S23 | "secondary care".ti,ab. |
| S24 | or/12-23 |
| S25 | ("personnel staffing" or "personnel scheduling" or "return to work").ti,ab. |
| S26 | "surge capacity".ti,ab. |
| S27 | exp emergency preparedness/ |
| S28 | "de-escalat*".ti,ab. |
| S29 | "relaxation*".ti,ab. |
| S30 | "return* to work".ti,ab. |
| S31 | "reset".ti,ab. |
| S32 | "new normal".ti,ab. |
| S33 | "post pandemic".ti,ab. |
| S34 | "pre pandemic".ti,ab. |
| S35 | "mitigat*".ti,ab. |
| S36 | "curtail*".ti,ab. |
| S37 | or/25-36 |
| S38 | exp Health Personnel/ |
| S39 | health workforce.mp. |
| S40 | "health* personnel".ti,ab. |
| S41 | "health* staff".ti,ab. |
| S42 | "health* workforce".ti,ab. |
| S43 | "health* worker*".ti,ab. |
| S44 | "medical staff".ti,ab. |
| S45 | "health* professional*".ti,ab. |
| S46 | "redeploy*".ti,ab. |
| S47 | or/38-46 |
| S48 | 11 and 24 and 37 and 47 |

*Appendix 1.4 – Search criteria: Web of Science*

| **Search number** | **Search terms** |
| --- | --- |
| S1 | ALL=(coronavirus infections) |
| S2 | ALL=(pneumonia) |
| S3 | ALL=(epidemic* OR pandemic*) |
| S4 | ALL=(coronavirus* OR coronovirus* OR coronaviral* OR coronoviral* OR coronavirinae* OR coronovirinae*) |
| S5 | ALL=(CoV) |
| S6 | ALL=("2019-nCoV" or "COVID-19" or "SARS-CoV-2" or "SARS-coronavirus-2") |
| S7 | ALL=(("respiratory* symptom*" or "respiratory disease*" or "respiratory illness*" or "respiratory condition*") AND (Wuhan* or Hubei* or China* or Chinese* or Huanan*)) |
| S8 | ALL=(("food market*") AND (Wuhan* or Hubei* or China*)) |
| S9 | ALL=((outbreak* or epidemic*) AND (Wuhan* or Hubei* or China* or Chinese*)) |
| S10 | ALL=("severe acute respiratory syndrome*") |
| S11 | ALL=("critical care") |
| S12 | ALL=("intensive care unit*" or "coronary care unit*" or "recovery room*" or "respiratory care unit*") |
| S13 | ALL=(“ICU”) |
| S14 | ALL=(“ITU”) |
| S15 | ALL=(“intensive care”) |
| S16 | ALL=("hospital*" or "general hospital*" or "high-volume hospital*" or "low-volume hospital*"or "public hospital*" or "teaching hospital*" or "urban hospital*" or "secondary care*" or "tertiary *care*") |
| S17 | ALL=("personnel staffing" or "personnel scheduling" or "return* to work") |
| S18 | ALL=("surge capacity") |
| S19 | ALL=("disaster planning") |
| S20 | ALL=("de-escalat*") |
| S21 | ALL=(reset) |
| S22 | ALL=("new normal") |
| S23 | ALL=("post pandemic") |
| S24 | ALL=("pre pandemic") |
| S25 | ALL=("curtail*") |
| S26 | ALL=("health* personnel") |
| S27 | ALL=("health* staff") |
| S28 | ALL=("health* workforce") |
| S29 | ALL=("health* worker*") |
| S30 | ALL=("medical staff") |
| S31 | ALL=(health* professional) |
| S32 | ALL=("redeploy*") |
| S33 | #10 OR #9 OR #8 OR #7 OR #6 OR #5 OR #4 OR #3 OR #2 OR #1 |
| S34 | #16 OR #15 OR #14 OR #13 OR #12 OR #11 |
| S35 | #25 OR #24 OR #23 OR #22 OR #21 OR #20 OR #19 OR #18 OR #17 |
| S36 | #32 OR #31 OR #30 OR #29 OR #28 OR #27 OR #26 |
| S37 | #36 AND #35 AND #34 AND #33 |

*Appendix 1.5 – Search criteria: Health Management Information Consortium/OVID*

| **Search number** | **Search terms** |
| --- | --- |
| S1 | exp Coronavirus/ |
| S2 | "coronavirus infection".ti,ab. |
| S3 | pneumonia/ |
| S4 | epidemics/ or pandemics/ |
| S5 | ((corona* or corono*) adj1 (virus* or viral* or virinae*)).ti,ab. |
| S6 | (coronavirus* or coronovirus* or coronavirinae* or CoV).ti,ab. |
| S7 | ("2019-nCoV" or "2019nCoV" or "nCoV2019" or "nCoV-2019" or "COVID-19" or "COVID19" or "CORVID-19" or "CORVID19" or "WN-CoV" or "WNCoV" or "HCoV-19" or "HCoV19" or "2019 novel*" or "Ncov" or "n-cov" or "SARS-CoV-2" or "SARSCoV-2" or "SARSCoV2" or "SARS-CoV2" or "SARSCov19" or "SARS-Cov19" or "SARSCov-19" or "SARS-Cov-19" or "Ncovor" or "Ncorona*" or "Ncorono*" or "NcovWuhan*" or "NcovHubei*" or "NcovChina*" or "NcovChinese*" or "SARS2" or "SARS-2" or "SARScoronavirus2" or "SARS-coronavirus-2" or "SARScoronavirus 2" or "SARS coronavirus2" or "SARScoronovirus2" or "SARS-coronovirus-2" or "SARScoronovirus 2" or "SARS coronovirus2").ti,ab. |
| S8 | (respiratory* adj2 (symptom* or disease* or illness* or condition*) adj5 (Wuhan* or Hubei* or China* or Chinese* or Huanan*)).ti,ab. |
| S9 | (("seafood market*" or "food market*" or "pneumonia*") adj5 (Wuhan* or Hubei* or China* or Chinese* or Huanan*)).ti,ab. |
| S10 | ((outbreak* or wildlife* or pandemic* or epidemic*) adj5 (Wuhan* or Hubei* or China* or Chinese* or Huanan*)).ti,ab. |
| S11 | "severe acute respiratory syndrome*".ti,ab. |
| S12 | or/1-11 |
| S13 | exp Critical Care/ |
| S14 | intensive care units/ or coronary care units/ or recovery room/ or respiratory care units/ |
| S15 | "ICU".ti,ab. |
| S16 | "ITU".ti,ab. |
| S17 | "intensive care".ti,ab. |
| S18 | "critical care".ti,ab. |
| S19 | hospitals/ or hospitals, general/ or hospitals, high-volume/ or hospitals, low-volume/ or hospitals, public/ or hospitals, teaching/ or hospitals, urban/ or secondary care centers/ or tertiary care centers/ |
| S20 | "tertiary healthcare".ti,ab. |
| S21 | "respiratory care unit".ti,ab. |
| S22 | hospital*.ti,ab. |
| S23 | "tertiary care".ti,ab. |
| S24 | "secondary care".ti,ab. |
| S25 | or/13-24 |
| S26 | exp Staffing levels/ or exp Patient nurse ratio/ |
| S27 | "surge capacity".ti,ab. |
| S28 | exp Emergency planning/ |
| S29 | "de-escalat*".ti,ab. |
| S30 | "relaxation*".ti,ab. |
| S31 | "return* to work".ti,ab. |
| S32 | "reset".ti,ab. |
| S33 | "new normal".ti,ab. |
| S34 | "post pandemic".ti,ab. |
| S35 | "pre pandemic".ti,ab. |
| S36 | "mitigat*".ti,ab. |
| S37 | "curtail*".ti,ab. |
| S38 | or/26-37 |
| S39 | health service staff/ |
| S40 | "health workforce".ti,ab. |
| S41 | "health* personnel".ti,ab. |
| S42 | "health* staff".ti,ab. |
| S43 | "health* workforce".ti,ab. |
| S44 | "health* worker*".ti,ab. |
| S45 | "medical staff".ti,ab. |
| S46 | "health* professional*".ti,ab. |
| S47 | "redeploy*".ti,ab. |
| S48 | or/39-47 |
| S49 | 12 and 25 and 38 and 48 |

*Appendix 1.6 – Search criteria: TRIP*

| **Search number** | **Search terms** |
| --- | --- |
| S1 | (healthcare workforce)(COVID-19)(de-escalate) (ICU) |

*Appendix 1.7 – Search criteria: NICE Evidence Search*

| **Search number** | **Search terms** |
| --- | --- |
| S1 | COVID 19 de-escalate ICU |

*Appendix 1.8 – Search criteria: medRxiv*

| **Search number** | **Search terms** |
| --- | --- |
| S1 | “de-escalate” AND "ICU" AND “COVID-19” AND “Health* workforce”" |

*Appendix 1.9 Justification of inclusion criteria*

We included a heterogenous range of literature based on empirical evidence from peer reviewed journals and grey literature. Opinion pieces that were not based on empirical evidence were excluded due to the need to prioritise factual evidence over non-factual evidence (70). The publication date was restricted to April 2021, with no starting restriction date, and the language was restricted to English. The population of interest for the review included healthcare staff that were returning from ICUs that they had been redeployed to during COVID-19. Publications were excluded if they discussed a broader hospital wide escalation and de-escalation process, without going into detail of the experiences within the ICUs specifically.

**Appendix 2 – Data extraction form**

| **Citation details** | - |
| --- | --- |
| **Type of publication** | Research article  Commentary article  Guidelines |
| **Study type** | Quantitative  Qualitative  Mixed methods  Other |
| **Study design** | Location of study  Timeframe  Aim of research |
| **Data collection** | Interviews  Observations  Focus groups  Surveys  Secondary data  Other |
| **Data analysis** | Content analysis  Thematic analysis  Quantitative analysis  Process mapping  Health needs assessment  Other |
| **Demography** | Professional group  Sample size |
| **Wellbeing strategies** | Counselling long term  Respect/ gratitude  Time off  Post-redeployment interviews |
| **Training strategies** | ICU training to continue  Emergency training to continue  Continued supervision  Catch up on missed training |
| **Operational strategies for staff** | Traffic light system  Change in rotas  Return to usual roles  Factor in time off  Retain staff  Change in staff hierarchy  Retain private providers |
| **Operational strategies for facilities** | Traffic light system  Changes in operational responsibility  Division of facilities  Ringfencing beds and resources |
| **Limitations of the study** | - |

**Appendix 3 – MMAT**

|  | **Poortaghi et al.** |
| --- | --- |
| **Screening questions** |  |
| Are there clear research questions? | Yes |
| Do the collected data allow to address the research questions? | Yes |
| **Qualitative studies** |  |
| Is the qualitative approach appropriate to answer the research question? | Yes |
| Are the qualitative data collection methods adequate to address the research question? | No |
| Are the findings adequately derived from the data? | Yes |
| Is the interpretation of results sufficiently substantiated by data? | Yes |
| Is there coherence between qualitative data sources, collection, analysis and interpretation? | Yes |
| **Quantitative descriptive studies** |  |
| Is the sampling strategy relevant to address the research question? | - |
| Is the sample representative of the target population? | - |
| Are the measurements appropriate? | - |
| Is the risk of nonresponse bias low? | - |
| Is the statistical analysis appropriate to answer the research question? | - |
| **Mixed methods studies** |  |
| Is there an adequate rationale for using a mixed methods design to address the research question? | - |
| Are the different components of the study effectively integrated to answer the research question? | - |
| Are the outputs of the integration of qualitative and quantitative components adequately interpreted? | - |
| Are divergences and inconsistencies between quantitative and qualitative results adequately addressed? | - |
| Do the different components of the study adhere to the quality criteria of each tradition of the methods involved? | - |
| **MMAT score** | 4/5 |

*For mixed methods studies is advised in the MMAT guidelines that ‘the overall quality of a mixed methods study cannot exceed the quality of its weakest component’ <http://mixedmethodsappraisaltoolpublic.pbworks.com/w/file/fetch/127916259/MMAT_2018_criteria-manual_2018-08-01_ENG.pdf>

**Appendix 4 – AACODS**

*Appendix 4.1 – ACCODS references 1-6*

|  | **Schneider et al.** | **Whitby et al.** | **Caroselli** | **Faculty of Intensive Care Medicine et al.** | **Price et al.** | **Marshall et al.** | **Leng et al.** |
| --- | --- | --- | --- | --- | --- | --- | --- |
| **Authority** | | | | | | | |
| Associated with a reputable organisation? | Yes | Yes | Yes | N/A | Yes | Yes | Yes |
| Professional qualifications or considerable experience? | Yes | Yes | Yes | N/A | Yes | Yes | Yes |
| Produced/published other work (grey/black) in the field? | Yes | Yes | Yes | N/A | Yes | Yes | Yes |
| Recognised expert, identified in other sources? | No | No | Yes | N/A | Yes | Yes | Yes |
| Cited by others? | Yes | N/A | Yes | N/A | Yes | Yes | Yes |
| Higher degree student under “expert” supervision? | N/A | N/A | N/A | N/A | N/A | N/A | N/A |
| Is the organisation reputable? | N/A | N/A | N/A | Yes | N/A | N/A | N/A |
| Is the organisation an authority in the field? | N/A | N/A | N/A | Yes | N/A | N/A | N/A |
| Does the item have a detailed reference list or bibliography? | Yes | Yes | Yes | No | Yes | Yes | Yes |
| Score | 1 | 1 | 1 | 1 | 1 | 1 | 1 |
| **Accuracy** | | | | | | | |
| Does the item have a clearly stated aim or brief? | No | No | No | No | No | Yes | Yes |
| If so, is this met? | N/A | N/A | N/A | N/A | N/A | Yes | Yes |
| Does it have a stated methodology? | No | No | No | No | No | Yes | Yes |
| If so, is it adhered to? | N/A | N/A | N/A | N/A | N/A | Yes | Yes |
| Has it been peer-reviewed? | No | No | No | No | No | No | Yes |
| Has it been edited by a reputable authority? | Yes | Yes | Yes | No | Yes | Yes | Yes |
| Supported by authoritative, documented references or credible sources? | Yes | Yes | No | No | Yes | Yes | Yes |
| Is it representative of work in the field? | Yes | Yes | Yes | Yes | Yes | Yes | Yes |
| If No, is it a valid counterbalance? | N/A | N/A | N/A | N/A | N/A | Yes | N/A |
| Is any data collection explicit and appropriate for the research? | Yes | Yes | N/A | N/A | N/A | Yes | Yes |
| If item is secondary material refer to the original. Is it an accurate, unbiased interpretation or analysis? | N/A | N/A | N/A | N/A | Yes | N/A | N/A |
| Score | 1 | 1 | 0 | 0 | 1 | 1 | 1 |
| **Coverage** | | | | | | | |
| Are any limits clearly stated? | Yes | Yes | Yes | Yes | Yes | Yes | Yes |
| Score | 1 | 1 | 1 | 1 | 1 | 1 | 1 |
| **Objectivity** | | | | | | | |
| Opinion, expert or otherwise, is still opinion: is the author’s standpoint clear? | Yes | Yes | Yes | No | No | No | Yes |
| Does the work seem to be balanced in presentation? | No | Yes | No | No | No | No | Yes |
| Score | 0.5 | 1 | 0.5 | 0 | 0 | 0 | 1 |
| **Date** | | | | | | | |
| Does the item have a clearly stated date related to content? | No | Yes | No | Yes | Yes | Yes | Yes |
| If no date is given, but can be closely ascertained, is there a valid reason for its absence? | No | N/A | Yes | N/A | N/A | N/A | N/A |
| Check the bibliography: have key contemporary material been included? | Yes | Yes | Yes | N/A | Yes | Yes | Yes |
| Score | 0 | 1 | 1 | 1 | 1 | 1 | 1 |
| **Significance** | | | | | | | |
| Is the item meaningful? (this incorporates feasibility, utility and relevance) | No | No | No | No | No | No | Yes |
| Does it add context? | Yes | Yes | Yes | No | Yes | Yes | Yes |
| Does it enrich or add something unique to the research? | Yes | Yes | Yes | Yes | Yes | Yes | Yes |
| Does it strengthen or refute a current position? | Yes | Yes | Yes | Yes | Yes | Yes | Yes |
| Would the research area be lesser without it? | Yes | Yes | Yes | Yes | Yes | Yes | Yes |
| Is it integral, representative, typical? | No | No | No | No | No | No | No |
| Does it have impact? | No | No | No | No | No | No | Yes |
| Score | 1 | 1 | 1 | 0 | 1 | 1 | 1 |
| **AACODS score** | **4.5/6** | **6/6** | **4.5/6** | **3/6** | **5/6** | **5/6** | **6/6** |

*Appendix 4.2 – AACODS references 7-12*

|  | **Yau et al.** | **McCabe et al.** | **Lord et al.** | **Panayiotou et al.** | **Lum et al.** | **Doyle et al.** | **Shaparin et al.** |
| --- | --- | --- | --- | --- | --- | --- | --- |
| **Authority** | | | | | | |  |
| Associated with a reputable organisation? | Yes | Yes | Yes | Yes | Yes | Yes | Yes |
| Professional qualifications or considerable experience? | Yes | Yes | Yes | Yes | Yes | Yes | Yes |
| Produced/published other work (grey/black) in the field? | Yes | Yes | Yes | Yes | Yes | Yes | Yes |
| Recognised expert, identified in other sources? | Yes | Yes | Yes | Yes | Yes | Yes | Yes |
| Cited by others? | No | Yes | Yes | No | No | No | No |
| Higher degree student under “expert” supervision? | N/A | No | N/A | N/A | N/A | Yes | N/A |
| Is the organisation reputable? | N/A | N/A | N/A | N/A | N/A | N/A | N/A |
| Is the organisation an authority in the field? | N/A | N/A | N/A | N/A | N/A | N/A | N/A |
| Does the item have a detailed reference list or bibliography? | Yes | Yes | Yes | Yes | Yes | Yes | Yes |
| Score | 1 | 1 | 1 | 1 | 1 | 1 | 1 |
| **Accuracy** | | | | | | |  |
| Does the item have a clearly stated aim or brief? | No | Yes | Yes | Yes | Yes | Yes | No |
| If so, is this met? | N/A | Yes | Yes | Yes | Yes | Yes | N/A |
| Does it have a stated methodology? | No | Yes | No | No | No | No | No |
| If so, is it adhered to? | N/A | Yes | N/A | N/A | N/A | N/A | N/A |
| Has it been peer-reviewed? | No | No | No | No | No | No | No |
| Has it been edited by a reputable authority? | Yes | Yes | Yes | Yes | Yes | Yes | Yes |
| Supported by authoritative, documented references or credible sources? | Yes | Yes | Yes | Yes | Yes | Yes | Yes |
| Is it representative of work in the field? | Yes | Yes | Yes | Yes | Yes | Yes | Yes |
| If No, is it a valid counterbalance? | N/A | N/A | N/A | N/A | N/A | N/A | N/A |
| Is any data collection explicit and appropriate for the research? | N/A | N/A | N/A | N/A | N/A | N/A | N/A |
| If item is secondary material refer to the original. Is it an accurate, unbiased interpretation or analysis? | N/A | N/A | N/A | N/A | N/A | N/A | N/A |
| Score | 0.5 | 1 | 1 | 1 | 1 | 1 | 0.5 |
| **Coverage** | | | | | | |  |
| Are any limits clearly stated? | Yes | Yes | Yes | Yes | Yes | Yes | Yes |
| Score | 1 | 1 | 1 | 1 | 1 | 1 | 1 |
| **Objectivity** | | | | | | |  |
| Opinion, expert or otherwise, is still opinion: is the author’s standpoint clear? | No | Yes | No | Yes | Yes | Yes | No |
| Does the work seem to be balanced in presentation? | No | No | Yes | Yes | No | No | No |
| Score | 0 | 0.5 | 0.5 | 1 | 0.5 | 0.5 | 0 |
| **Date** | | | | | | |  |
| Does the item have a clearly stated date related to content? | Yes | Yes | Yes | Yes | Yes | Yes | Yes |
| If no date is given, but can be closely ascertained, is there a valid reason for its absence? | N/A | N/A | N/A | N/A | N/A | N/A | N/A |
| Check the bibliography: have key contemporary material been included? | No | Yes | Yes | No | Yes | Yes | Yes |
| Score | 0.5 | 1 | 1 | 0.5 | 1 | 1 | 1 |
| **Significance** | | | | | | |  |
| Is the item meaningful? (this incorporates feasibility, utility and relevance) | No | No | No | No | No | No | No |
| Does it add context? | Yes | Yes | Yes | Yes | Yes | Yes | Yes |
| Does it enrich or add something unique to the research? | Yes | Yes | Yes | Yes | Yes | Yes | Yes |
| Does it strengthen or refute a current position? | Yes | Yes | Yes | Yes | Yes | Yes | Yes |
| Would the research area be lesser without it? | Yes | Yes | Yes | Yes | Yes | Yes | Yes |
| Is it integral, representative, typical? | No | No | No | No | No | No | No |
| Does it have impact? | No | No | No | No | No | No | No |
| Score | 1 | 1 | 1 | 1 | 1 | 1 | 1 |
| **AACODS score** | **4/6** | **5.5/6** | **5.5/6** | **5.5/6** | **5.5/6** | **5.5/6** | **4.5/6** |

AACODS checklist: <https://canberra.libguides.com/c.php?g=599348&p=4148869>
